# Metabolites of alectinib in human: their identification and pharmacological activity

**DOI:** 10.1016/j.heliyon.2017.e00354

**Published:** 2017-07-10

**Authors:** Mika Sato-Nakai, Kosuke Kawashima, Toshito Nakagawa, Yukako Tachibana, Miyuki Yoshida, Kenji Takanashi, Peter N. Morcos, Martin Binder, David J Moore, Li Yu

**Affiliations:** a Research division, Chugai Pharmaceuticals, Co., Ltd., 1-135 Komakado, Gotemba, Shizuoka 412-8513, Japan; b Roche Innovation Center New York, 430 East 29th Street, New York, NY10016, United States; c Roche Innovation Center Basel, Knozern-Hauptsitz, Grenzacherstrasse 124, CH-4070, Basel, Switzerland

**Keywords:** Metabolism, Cancer research, Pharmaceutical science

## Abstract

Two metabolites (M4 and M1b) in plasma and four metabolites (M4, M6, M1a and M1b) in faeces were detected through the human ADME study following a single oral administration of [^14^C]alectinib, a small-molecule anaplastic lymphoma kinase inhibitor, to healthy subjects. In the present study, M1a and M1b, which chemical structures had not been identified prior to the human ADME study, were identified as isomers of a carboxylate metabolite oxidatively cleaved at the morpholine ring. In faeces, M4 and M1b were the main metabolites, which shows that the biotransformation to M4 and M1b represents two main metabolic pathways for alectinib. In plasma, M4 was a major metabolite and M1b was a minor metabolite. The contribution to *in vivo* pharmacological activity of these circulating metabolites was assessed from their *in vitro* pharmacological activity and plasma protein binding. M4 had a similar cancer cell growth inhibitory activity and plasma protein binding to that of alectinib, suggesting its contribution to the antitumor activity of alectinib, whereas the pharmacological activity of M1b was insignificant.

## 1 Introduction

Non-small cell lung cancer (NSCLC) is a leading cause of cancer-related mortality worldwide. Survival rates for lung cancer tend to be much lower than for other common cancers as a result of late diagnosis and limited effective therapy in the advanced stages of the disease [1]. In 6.7% of metastatic NSCLC patients, the echinoderm microtubule-associated protein-like 4(*EML4*)-anaplastic lymphoma kinase (*ALK*) fusion transcript was detected in Japan [2, 3]. Although an *ALK* inhibitor, crizotinib was developed [4], resistance to the treatment was reported [5]. For the treatment of patients with *ALK*-positive unresectable, recurrent/advanced NSCLC, a small-molecule *ALK* inhibitor, alectinib (chemically identified as 9-ethyl-6,6-dimethyl-8-(4-morpholino-1-piperidyl)-11-oxo-5*H*-benzo[*b*]carbazole-3-carbonitrile, Alecensa^®^) [6, 7], was approved by the Ministry of Health, Labour and Welfare in Japan in July, 2014. It was granted accelerated approval by the Food and Drug Administration in the United States in December 2015, and was conditionally approved by the European Commission in February 2017.

In the early development phase of drugs, it is essential to predict the metabolites in human plasma and elucidate the main metabolic pathway. In general, hepatocytes and liver subcellular fractions are commonly known and predictive approaches to elucidate these steps have been published [8, 9, 10, 11], however, these *in vitro* approaches have proved insufficient to reflect the *in vivo* profile and the main metabolic pathway in human in the case of such as slow metabolite turnover rate, and extrahepatic metabolism. Therefore, a human ADME study is essential to determine via mass balance the elimination pathways of an administered drug and give a metabolite profile definitively in human.

In the case of alectinib, *in vitro* hepatocytes and *in vivo* rat studies revealed M4　(9-ethyl-8-[4-(2-hydroxyethylamino)-1-piperidyl]-6,6-dimethyl-11-oxo-5*H*-benzo[*b*]carbazole-3-carbonitrile) to be the major metabolite, and M6 (8-(4-amino-1-piperidyl)-9-ethyl-6,6-dimethyl-11-oxo-5*H*-benzo[*b*]carbazole-3-carbonitrile), and the uncharacterized metabolite M1 to be the minor metabolites in plasma and in hepatocytes (Nakagawa et al., Xenobiotica, in press), and the elimination pathway in rats was elucidated [12].

Subsequently, a human ADME study following an oral administration of 600 mg/67 μCi [^14^C]alectinib was conducted and the results have been reported by Morcos et al. [13]. Here we report how we determined the main metabolic pathway and evaluated the pharmacological relevance of identified metabolites in human.

## 2 Materials and methods

### 2.1 Materials

**Fig. 1:**
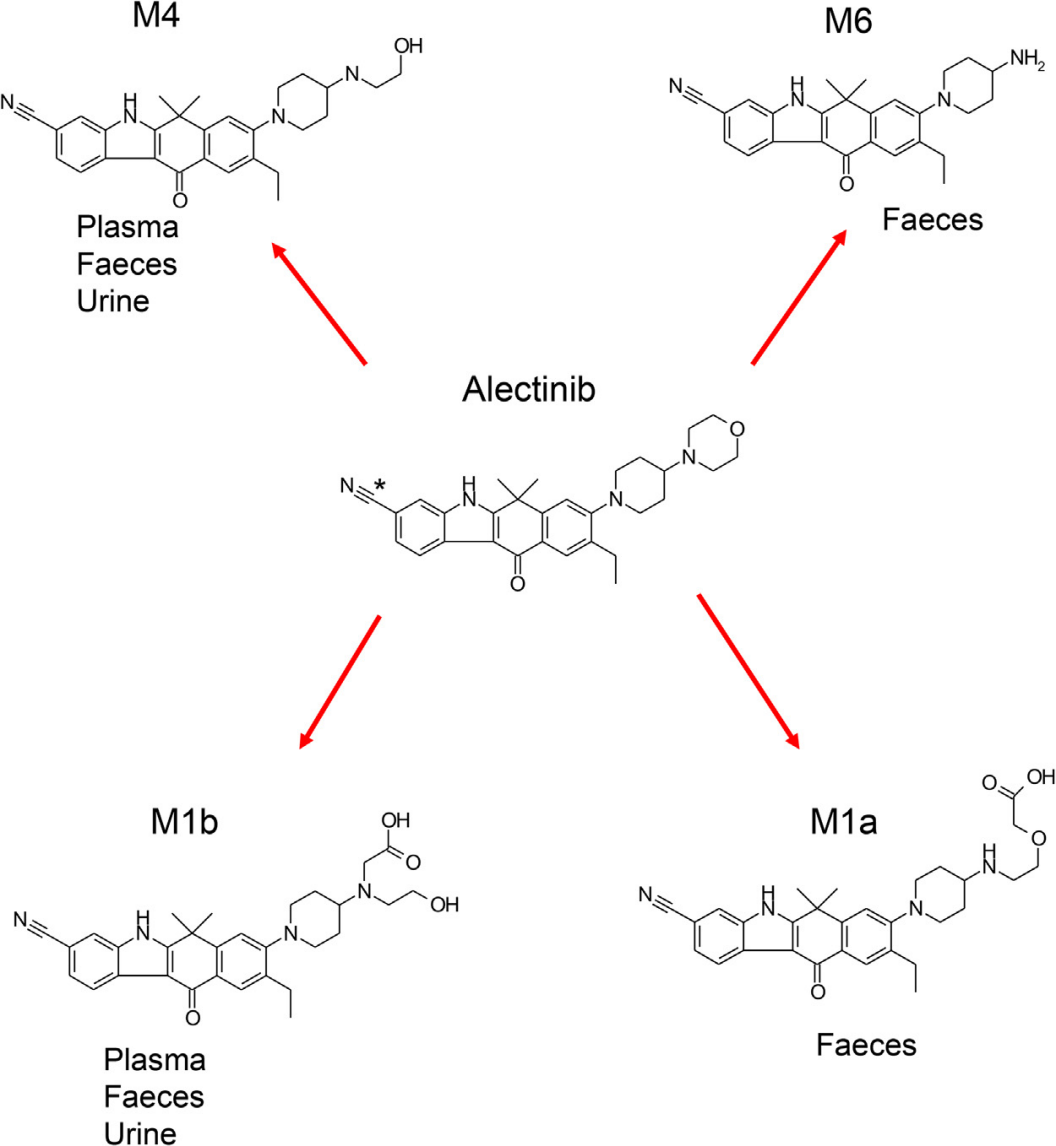
Proposed metabolic pathways of alectinib, showing structures of alectinib with position of [^14^C] shown by an asterisk.

Alectinib (PubChem CID: 49806720) and all its metabolites, M1a, M1b, M4 (PubChem CID: 66835300), and M6 (PubChem CID: 66834636)) including a stable isotope, were synthesised in Chugai Pharmaceuticals, Co., Ltd. (Tokyo, Japan). [^14^C]alectinib indicated in Fig. 1 was synthesised in PerkinElmer Co., Ltd. (Boston, MA). The chemical purities of these compounds were 98.4% for [^14^C]alectinib (radio-HPLC), 100.0% for alectinib (UV-HPLC), 98.4% for M4 (UV-HPLC), 98.6% for M6 (UV-HPLC), 95.4% for M1a (UV-HPLC), and 94.7% for M1b (UV-HPLC), respectively.

### 2.2 Samples

Samples for metabolite profiling and identification were obtained from the human ADME study after dosing 600 mg/67 μCi [^14^C]alectinib to six healthy male subjects [13]. Human plasma for protein binding was obtained from three healthy male volunteers. All participants provided written informed consent prior to study related procedures. The protocol of human ADME study was approved by the Independent Ethics Committee of the Stichting Beoordeling Ethiek Bio-Medisch Onderzoek (Assen, The Netherlands), and that of protein binding study was approved by Chugai IRB.

#### 2.2.1 Plasma

Plasma samples were pooled at 4 h and 6 h (4 h + 6 h), and 8 h and 12 h (8 h +12 h) across the six subjects [13]. An 18-mL aliquot of pooled plasma was extracted with the same volume of acetonitrile twice, and then the extract was lyophilized. The resulting residue was further extracted with 3 mL of acetonitrile/water (9:1, v/v) twice and lyophilized again. The resulting residue was reconstituted with 900 μL of methanol/water (7:3, v/v) to obtain the supernatant as an HPLC sample in which plasma was 20-fold concentrated.

The HPLC sample (300 μL) was weighed and its radioactivity measured using Equation 1: Recovery of radioactivity (%) = {Radioactivity concentration in extract or HPLC sample (dpm/g) × Whole sample weight (g)}/Radioactivity in pooled sample (dpm) × 100.

#### 2.2.2 Urine

Urine samples were pooled at 0–24 h, and at 24–72 h across the six subjects [13]. A 16-mL aliquot of pooled urine was concentrated 20-fold using the same process for plasma.

#### 2.2.3 Faeces

Faecal samples were pooled at 0–48 h, 48–96 h, and 96–168 h across the six subjects [13]. One to two weight equivalents of water was added to the faecal samples and the samples were homogenized and mixed with the same volume of acetonitrile. After following the same procedure as for plasma, the residue was reconstituted with methanol/water (7:3, v/v) or dimethyl sulfoxide (DMSO) and　centrifuged, and the faecal sample was concentrated 5-fold for use in analysis.

### 2.3 Analysis

#### 2.3.1 HPLC conditions

The following two conditions for HPLC analysis using the L-2000 series with UV detector L-2400 (Hitachi High-Technologies Corp.) were used in this study, with the acidic conditions applied only when separating M1 metabolites.

Alkaline Conditions: For metabolite profiling, the extracted samples from plasma, urine, and faeces were subjected to HPLC under alkaline conditions using XBridge™ Shield RP18, 3.5 μm, 4.6 × 150 mm (Waters), and a linear stepwise gradient with solvent A (consisting of water/30%trimethylamine at 100:0.1, v/v) and solvent B (consisting of acetonitrile/30%trimethylamine at 100:0.1, v/v), and a flow rate of 1 mL/min. Solvent B in the gradient started at 1% and then changed as follows: 30% (2 min), 60% (29 min), 98% (29.1 min), 98% (32 min), 1% (32.1 min), and 1% (40 min). The UV wavelength was set at 335 nm.

Acidic Conditions: For separation and identification of the metabolites M1a and M1b, acidic conditions were applied using a Sunniest C18, 3 μm, 4.6 × 150 mm (ChromaNik Technologies Inc.), and a linear stepwise gradient with solvent A (consisting of 98% formic acid/water at 0.1:100, v/v) and solvent B (consisting of formic acid/acetonitrile at 0.1:100, v/v), and the flow rate was set to 1 mL/min. Solvent B in the gradient started at 5% and was then changed as follows: 40% (20 min), 95% (30 min), 98% (35 min), 5% (35.1 min), and 5% (40 min). The UV wavelength was set at 230–400 nm.

#### 2.3.2 Metabolite profiling

Plasma and Urine: To profile metabolites, 150 μL of the same HPLC sample of plasma or 100 μL of the same HPLC sample of urine was injected 3 separate time, and the eluates collected at 15-second intervals were combined to be measured by a liquid scintillation counter (LSC) (Tri-Carb 3100TR, PerkinElmer, Inc.) at a low level count mode for 10 min.

Faeces: A radiodetector (Radiomatic 625TR, PerkinElmer, Inc.) was used, and the area percentage of each radioactive peak on the radiochromatograms was calculated with analysis software (FLO-ONE Ver. 3.65, PerkinElmer, Inc.).

#### 2.3.3 HPLC-Mass Spectrometer (LC-MS) analysis

The HPLC samples from all matrixes were injected into the LC-MS system (Accela-LTQ Orbitrap XL, Thermo Fisher Scientific K.K.) with electrospray ionization (ESI), in negative mode using the following settings: MS, Fourier transformation (FT, resolution: 30000); MS^2^, higher energy collisional dissociation; sheath gas flow rate, 40 arb: aux gas flow rate, 5 arb: spray voltage, 5.00 kV; capillary voltage, −13.00 V; capillary temperature, 350 °C; collision energy, 130; and tube Iens, −163.12 V.

The M1 peak in the faeces samples from 48–96 h was fractionated by HPLC under alkaline conditions and reconstituted with methanol/water (7:3, v/v). The sample was applied to LC-MS under acidic conditions with ESI, in positive mode and the following settings; MS, Fourier transformation (FT, resolution: 30000); MS^2^, higher energy collisional dissociation; sheath gas flow rate, 60 arb: aux gas flow rate, 20 arb: spray voltage, 5.00 kV: capillary temperature, 350 °C; collision energy, 45; and tube lens, 130.00 V.

#### 2.3.4 Nuclear magnetic resonance (NMR)

Samples prepared for HPLC from the pooled faecal samples taken at 48–96 h were injected as 100 μL aliquots into the HPLC system under alkaline conditions repeatedly (70 times) to collect M1. Subsequently, using acidic conditions, 100-μL aliquots of the collected fraction were injected into the HPLC system 18 times to collect the major component (M1b). The M1b fraction was evaporated under a nitrogen stream and dissolved in 40 μL-DMSO-*d6* to prepare the NMR sample. Spectra were taken on a Bruker 500 MHz Avance III spectrometer equipped with a cryogenic 1.7 mm TCI probe head at a temperature of 305 K. Spectrometer operations and data processing were done using Topspin 3.0 (Bruker, Fällanden). The ^1^H and ^13^C chemical shifts were referenced to the residual solvent signals of DMSO at δ 2.50 ppm and 40.3 ppm, respectively. Experiments performed were ^1^H-NMR, ^1^H-^13^C HSQC (Hetero-nuclear Single Quantum Coherence), ^1^H-^13^C HMBC (Hetero-nuclear Multiple-Bond Connectivity), ^1^H-COSY (C Orrelation SpectroscopY), ^1^H-ROESY (Rotating-frame Overhauser SpectroscopY), ^1^H-^15^N HMBC.

### 2.4 *In vitro* pharmacological activity

The inhibitory activity of alectinib metabolites against ALK was measured by quantitatively analysing the phosphorylation of substrate peptides by each recombinant enzyme protein in the presence of test articles, using a europium-labelled anti-phospho-substrate antibody. Solutions of test articles, substrate/ATP (2 μmol/L substrate peptide-0.2 mol/L HEPES buffer containing 60 μmol/L ATP, 20 mmol/L MgCl_2_, 0.2 mmol/L DTT, and 40 μmol/L Na_3_VO_4_) and the enzyme (final concentration of 200 ng/mL) were mixed in a clear-bottomed 384-well black plate and incubated for 90 min at 30 °C. After adding stop solution (100 mmol/L EDTA-0.1 mol/L HEPES buffer), TR-FRET reagent (2.5 nmol/L of LANCE^®^ Eu-W1024-labeled anti-phosphotyrosine (PT66) and 6.25 μg/mL of Streptavidine SureLight^®^ APC) was added and the time-resolved fluorescence was measured by EnVision HTS (Model 2104, PerkinElmer).

The inhibitory activity by alectinib metabolites on cell growth was measured by quantitatively analysing the ATP content in cells treated with the test articles, using CellTiter-Glo^®^ Luminescent Cell Viability Assay (Promega). The NCI-H2228 NSCLC cell line that harbours *EML4-ALK* fusion was seeded onto a PrimeSurface spheroid 96U plate (Sumitomo Bakelite) at 2 × 10^3^ cells/well and cultured in a CO_2_ incubator (37 °C, 5% CO_2_) overnight. Then test article solutions were added and the cell viability was measured after a 120-h incubation in a CO_2_ incubator. The luminescence was measured by EnVision HTS and the cell viability was calculated.

### 2.5 Plasma protein binding

Plasma was obtained by centrifugation (4 °C, 1600 *g*, 10 min, CF7D2, Hitachi Koki) of EDTA‐treated blood from three healthy male volunteers and was then pooled. The plasma was spiked with a portion of [^14^C]alectinib or M4 solution (final 0.5% DMSO) and was added to the donor side of an equilibrium dialyzer (equilibrium dialyzer cell EC-1, Sanplatec) set with a Spectra/Por 2 membrane (Spectrum Laboratories) and dialyzed against the same volume of PBS added to the receiving side at 37 °C for 24 h. An aliquot of the plasma or PBS was taken after incubation, and the radioactivity was measured by LSC (TRI-CARB 2300TR, PerkinElmer) for [^14^C]alectinib. Samples for M4 were mixed with deuterated M4 as an internal standard and acetonitrile/methanol (1:1, v/v). After centrifugation, the supernatant was injected into an LC/ESI-MS/MS system consisting of ACQUITY Ultra Performance LC (Waters) and API 4000 (AB Sciex) in positive-ion multiple-reaction monitoring (MRM) mode to measure M4 (Q1: m/z 457 and Q3: m/z 396) concentration. Chromatography was performed using ACQUITY UPLC BEH Phenyl (2.1 mm I.D. × 50 mm, 1.7 μm, Waters) at 60 °C; MP A: water/98% formic acid (1000/1, v/v), MP B: methanol/98% formic acid (1000/1, v/v), with a flow rate of 0.7 mL/min and a gradient program as follows: (time, % MP B): 0 min, 40%; 0.3 min, 40%; 1.9 min, 45%; 1.91 min, 90%; 2.20 min, 90%; 2.21 min, 40%; 2.70 min, stop. For the protein binding of M4, 1–5000 ng/mL in human plasma, and 0.5–2500 ng/mL in PBS were calibrated.

The recovery was calculated by the following equation.

Recovery = (Cf + Cp)/(actual concentration of alectinib or M4 in plasma before dialysis) x 100 (%)

Protein binding was calculated by the following equation.

Protein binding = (1−Cf/Cp) × 100 (%)

Where Cf and Cp represent the concentration in PBS and in plasma, respectively.

## 3 Results

### 3.1 Metabolite profiling

The levels of radioactivity recovered in plasma, urine, and faeces extracts after sample processing were 83%–85%, 91%–92%, and 74%–86%, respectively, and these levels were sufficiently high to use in metabolite profiling.

As summarized in Table 1 [13], which shows the percentages of drug-related compounds, M4 and M1 were the only metabolites detected in plasma. However, of all metabolites identified in faecal samples (M1, M4, and M6), M1 and M4 were found to be the most abundant (7.2% and 5.8% of the dose administered, respectively [13]). M1 was the most abundant metabolite in urine, with a recovery ratio accounting for approximately 0.5% of the dose administered.

**Table 1:** Percentage of drug-related compounds in plasma, urine, and faeces samples following a single oral administration of [^14^C]alectinib.

Drug-related compound	Percentage of drug related compound in analytical sample (%)						
	Plasma		Urine		Faeces		
	4 h + 6 h	8 h + 12 h	0–24 h	24–72 h	0–48 h	48–96 h	96–168 h
Alectinib	85.2	90.1	ND	ND	95.0	76.5	23.0
M1	7.8	ND	91.0	75.8	3.4	11.5	34.1
M4	7.0	9.9	9.0	24.2	1.1	11.0	37.3
M6	ND	ND	ND	ND	ND	ND	5.6

ND: Not detected.

Arranged from Table by Morcos et al. [13]. Samples were pooled across subjects (n = 6) at indicated time points for metabolite profiling. The recovery of radioactivity within excretion up to 168 h post-dose was 97.8% in faeces and 0.5% in urine.

### 3.2 Identifying the structure of M1

**Fig. 2:**
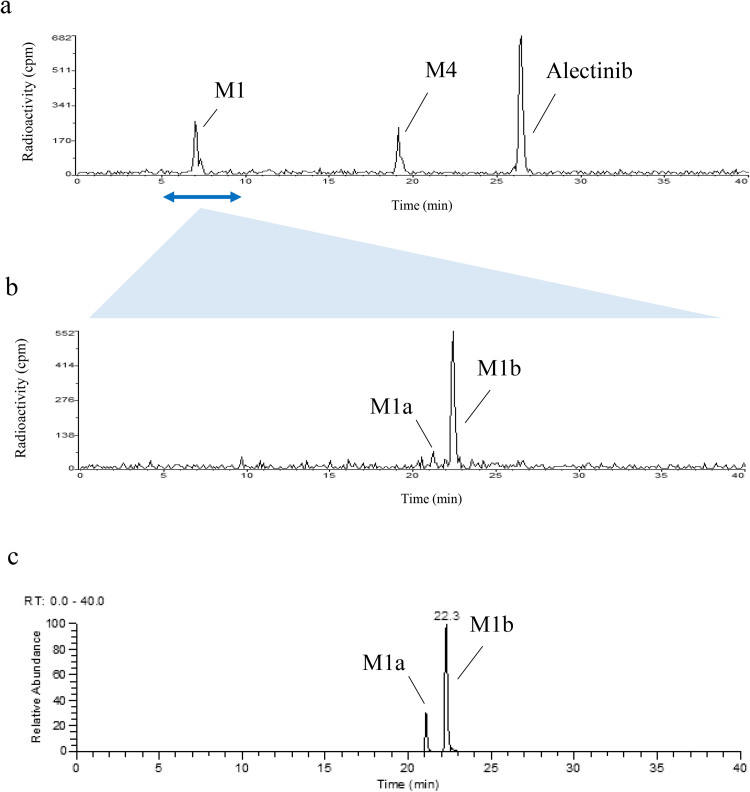
Chromatograms for (a) radioactivity of pooled faecal homogenate (48–96 h) under alkaline conditions, and (b) radioactivity and (c) extracted ion at m/z 515.26528 ± 5 ppm of fractionated M1 under acidic conditions.

**Fig. 3:**
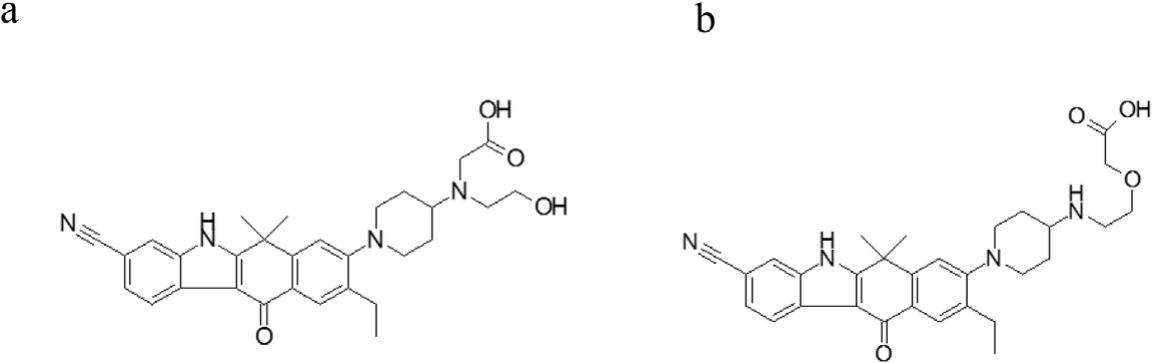
Two candidate metabolite structures in M1.

**Fig. 4:**
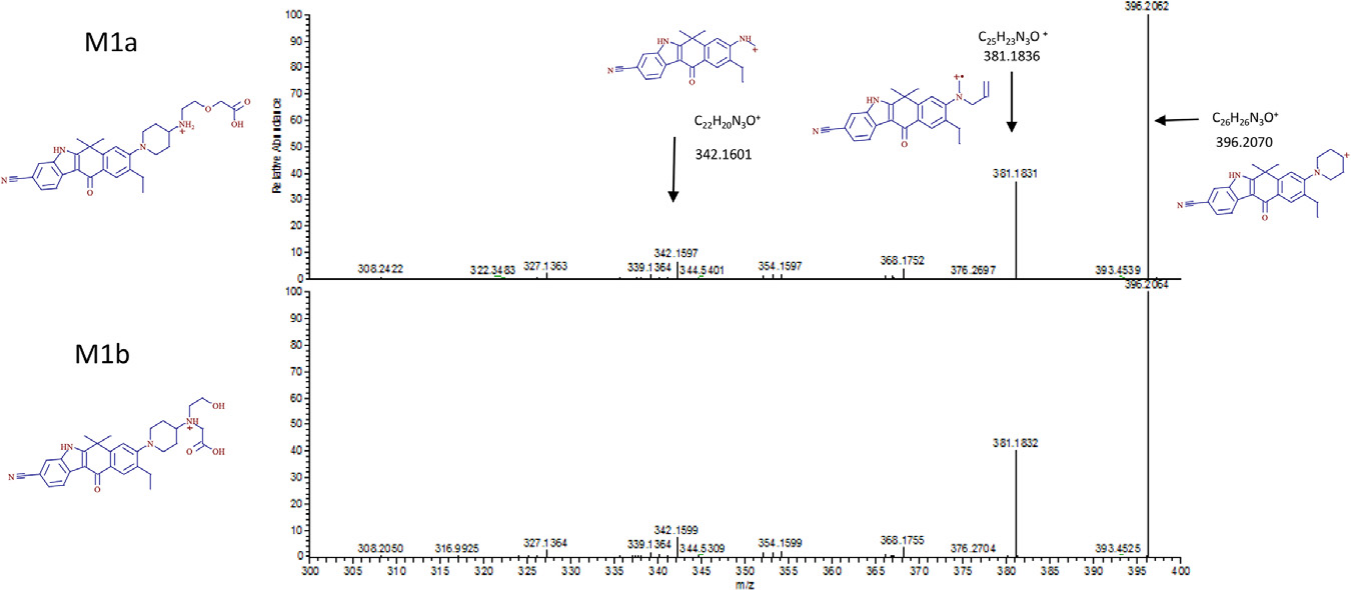
ESI-positive MS/MS spectra of M1a and M1b separated under acidic conditions from the faecal sample from 48 h to 96 h, and proposed structures of the fragments.

From an examination of the radiochromatogram shown in Fig. 2a and an ESI-negative/MSMS spectrum of M1 at m/z 513.25073 ± 5 ppm (data not shown), which indicated a 32Da addition to alectinib under alkaline conditions, the metabolite structure was predicted to be a carboxylate metabolite oxidatively cleaved at the morpholine ring, and two carboxylate structures were assumed, as shown in Fig. 3. To determine the main component of M1, metabolite separation was optimized by applying acidic HPLC conditions after the alkaline HPLC conditions. Acidic conditions alone or applied prior to alkaline conditions cannot separate metabolites well because the larger amount of alectinib forms a peak that interferes with those of other metabolites. Therefore, the M1 peak was subsequently injected into the HPLC-radiodetector and MS system under acidic conditions. As shown in Fig. 2b, two peaks were detected in the radiochromatogram: a smaller peak at the retention time (t_R_) of ∼21 min (M1a) and a larger peak at t_R_ of ∼22 min (M1b). The ratio of the area of the M1a peak to the M1b peak was approximately 1 to 9 in the radiochromatogram (Fig. 2b), which confirmed that M1 was a mixture of two metabolites M1a and M1b, and defined M1b as the main component. On the extracted ion chromatogram (XIC) of the protonated molecule ([M + H]^+^, m/z 515.26528 ± 5 ppm), the ratio of the two peaks detected as M1a and M1b was approximately 1 to 3, as shown in Fig. 2c. Moreover, their product ions were identical (Fig. 4), so M1a and M1b were considered to be isomers.

**Fig. 5:**
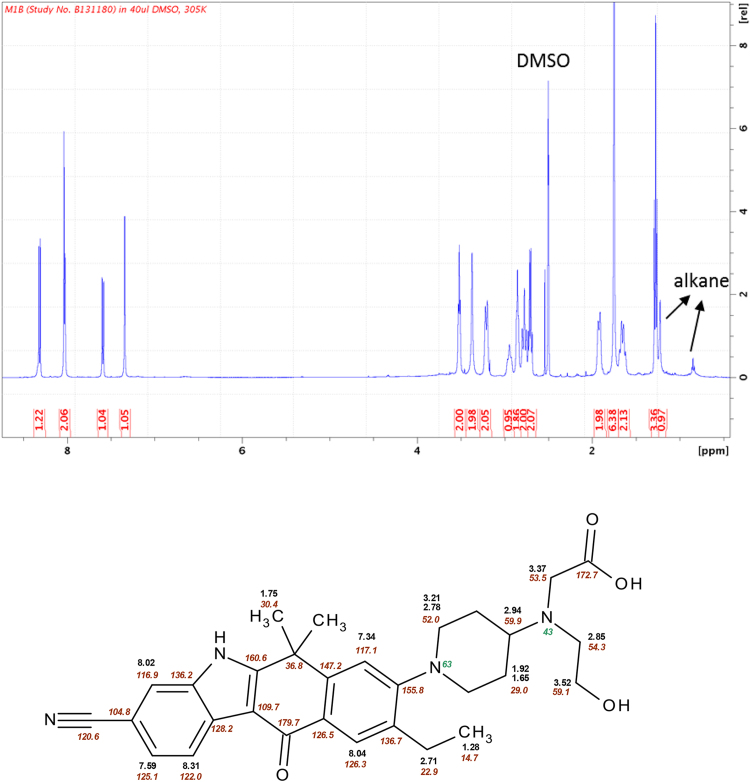
1H-NMR spectrum and the structure of M1b with chemical shifts of protons, carbons, and nitrogens.

To identify the structure of M1b, NMR was carried out. After inspecting the ^1^H-NMR (Fig. 5) and the HSQC spectrum taken in ^1^H,^13^C, it was obvious that the aromatic part of the molecule was unaffected because chemical shifts of proton and carbon were very similar to those of the parent molecule and the piperidine moiety also showed only minor differences compared to the parent molecule. Instead of the four cross-peaks of the morpholine moiety, only three CH_2_ groups (3.37/53.5 ppm, 3.52/59.1 ppm, 2.85/54.3 ppm) were visible in the HSQC spectrum, two of which (3.52 ppm, 2.85 ppm (triplets)) were linked together in the COSY spectrum (data not shown). An examination of the ^1^H,^13^C HMBC spectrum (data not shown) revealed a strong correlation from proton 3.37 ppm to a carbon with a chemical shift of 172.7 ppm, which is very characteristic of a carbon in a carbonyl group. The proton signal 3.37 ppm showed two additional correlations to carbons with chemical shifts of 54.3 ppm and 59.9 ppm. The proton of the CH group of piperidine in a ROESY spectrum showed cross-peaks to the following protons: 3.52 ppm, 3.37 ppm, 2.85 ppm, 2.78 ppm, and 1.92 ppm. The nitrogen of the former morpholine group has a chemical shift of 43 ppm, which also fits very well as an amine. Summarising all NMR data on chemical shifts (^1^H, ^13^C, ^15^N), and 2D correlations, it was evident that the morpholine ring was oxidatively opened, at which a carboxyl group developed, and thus the structure of M1b, as shown in Fig. 3a, was homologised directly from the human faecal sample. Based on this result, M1b and its isomer M1a were chemically synthesised and the minor component of M1a was identified by LC-MS analysis.

Plasma and urine samples were subjected to the LC‐MS system under acidic conditions. The each t_R_ of XIC and their product ions of M1a and M1b in all matrices under acidic conditions were identical. The ratio of M1a to M1b was similar between all matrices (plasma: 1/8, urine: 1/10 in the XIC). Therefore, M1a and M1b were identified, and M1b was the major metabolite in M1 in all matrices.

### 3.3 *In vitro* pharmacological activity

*In vitro* pharmacological activity of all circulating metabolites is summarized in Table 2. In the cell-free assay, the metabolites exhibited similar potent inhibitory activity against ALK, with IC_50_ values ranging from 1.2 to 1.9 nmol/L. On the other hand, their inhibitory activity against the growth of NCI-H2228 cells, which harbours *EML4-ALK*, was markedly different. M4 inhibited the cell growth with an IC_50_ value of 37 nmol/L, similar to alectinib (33 nmo/L [7]), whereas M1b showed a much weaker effect.

**Table 2:** In vitro inhibitory activity of alectinib metabolites against *ALK* or tumour cell growth.

Metabolite	IC_50_ (nmol/L)	
	ALK	Cell growth
Alectinib*	1.9	33
M1b	1.6	320
M4	1.2	37

Values represent the geometric mean of triplicates.

* This value is referred [7].

### 3.4 Plasma protein binding

Alectinib and M4 showed similar high binding to human plasma protein as shown in Table 3. The binding was higher than 99% at the range of concentration investigated and did not depend on the concentration. The recoveries of alectinib and M4 were 85.0 ± 1.7% and 82.7 ± 2.5%, respectively.

**Table 3:** Plasma protein binding of alectinib and M4.

	Concentration	Protein Binding
	(μg/mL)	(%)
Alectinib	0.1	99.6 ± 0.1
	1	99.7 ± 0.0
	10	99.7 ± 0.0
M4	0.3	99.1 ± 0.1
	1	99.5 ± 0.0
	2	99.5 ± 0.1

Values represent the mean ± standard deviation of triplicates.

## 4 Discussion

After oral administration of [^14^C]alectinb at 600 mg, the main elimination route for alectinib was faeces (97.8% of dose administered within 168 hours) in which unabsorbed alectinib was the most abundant component followed by two metabolites, M4 and M1 [13]. This study describes the methods that enabled all metabolites observed in human to be profiled and characterised.

The chemical structure of M1 had not been identified in preclinical studies prior to the human ADME study. Generally, biosynthesised approaches that use human or animal tissues, microorganisms, or genome mutation are useful for identifying human metabolites [14, 15]. However, because the *in vitro* metabolite turnover rate of alectinib was low, identifying M1 directly from human *in vivo* samples was considered to be the most efficient way.

Peak shapes in the initial *in vitro* metabolite profiling study in human hepatocytes　suggested that M1 was composed of multiple components. The shorter retention time and MS/MS spectrum of HPLC-MS under alkaline conditions in human provided further evidence that the possible metabolites were two isomers of a carboxylate, which is generally known as a metabolite of a morpholine moiety [16, 17]. When different HPLC conditions were applied to separate the multiple components, the major component M1b was successfully separated from the minor component M1a using an acidic mobile phase. The chemical structure decided by NMR from an isolated faecal sample showed that it was, as predicted, a carboxylate metabolite oxidatively cleaved at the morpholine ring. The results of this study confirmed M1 to be largely composed of M1b with negligible contribution by M1a. All metabolites of alectinib in human detected by radioactivity had been completely identified (Fig. 1).

The results show that all metabolites of alectinib go through morpholine ring opening, which has been reported *in vivo* for other drugs [16, 17, 18, 19]. The oxidation site in the morpholine ring depends on the drugs, as does the relative proportion of these pathways, which is also reported to be affected by species difference [20]. In the case of alectinib, M1b was the major component of M1 in human, but M1a was larger than M1b in the bile in rats, when confirmed after the human ADME study (in-house data); however the mechanism of the species difference was unclear. For the human ADME study, dosimetry calculation was used to decide the appropriate radiation burden from data on tissue distribution and mass balance obtained in rats [12]. Alectinib, with logD 1.96 at pH 3.575 and pKa 7.05 [21], could bind melanin similarly to many lipophilic basic drugs [22, 23]. The dosimetry calculations limited alectinib to 2.5 MBq (67 μCi)/body and the specific radioactivity of a clinical dose of 600 mg was calculated as 4.17 MBq/g. For the alectinib human ADME study, [^14^C]alectinib at C_max_ was 175 ng/mL [13], or approximately 40 dpm/mL, but generally, the limit of detection of metabolite profiling by LC-LSC is approximately 100 dpm/mL. Alectinib’s low plasma radioactivity made it necessary to find a method with sufficient sensitivity to appropriately evaluate a metabolite at a level of 10% of the total radioactivity in plasma. An ultra-sensitive LSC method, using three successive injections of plasma samples concentrated 20-fold was devised, which made it possible to detect levels lower than 1 dpm/mL, and M4 and M1 were detected in plasma in the human ADME study. Ion intensity in the XIC of the protonated molecular ion revealed that M1 was a mixture of M1a and M1b with a relative abundance ratio for M1a/M1b of 1/8 in plasma; however, when the ratios for the M1 peaks isolated from the human faecal sample determined by radioactivity (1/9) were compared with those found in the XIC (1/3), it was evident that the MS ratio for M1a/M1b was higher than the real abundance ratio. Therefore, M1b was determined as the main component of M1, and M1a was considered negligible in plasma. In addition, the AUC ratios for the percentage of total drug-related systemic exposure of alectinib and M4 were 61% and 15%, respectively, when standard calibration on LC/MS was used to measure drug concentrations and total radioactivity in plasma [13]. Therefore, although M1b circulated in plasma, it was considered a minor metabolite because of its transient appearance (only detected in the 4 h + 6 h plasma sample) and its detection relative to M4.

When the pharmacological activity of the two circulating metabolites (M4 and M1b) was assessed in a cell-free assay, alectinib, M4, and M1b each exhibited similar potent inhibitory activity against ALK, but in the cell-based assay, M4 demonstrated similar inhibitory activity to alectinib, while M1b demonstrated little relevant pharmacological activity. The 20–30 fold shift in IC_50_s for alectinib and M4 from the ALK enzyme inhibition assay to the cell growth inhibition assay may be mostly contributed by the protein binding in the cell medium which contained 10% FBS. However the very poor cellular activity of M1b (∼200-fold IC_50_ shift) is probably due to both its protein binding and lower membrane permeability because M1b has lower lipophilicity (LogD = 0.356, Pipeline pilot 9.1, Biovia) than M4 (LogD = 3.235, Pipeline pilot 9.1) and, as a carboxylic, has a negative charge. Therefore, the low circulating exposure and negligible pharmacological activity of M1b indicated that it did not contribute to alectinib activity *in vivo*.

As the M4-to-alectinib AUC ratio is ∼50% [13], and M4 and alectinib had similar *in vitro* potency and protein binding (human plasma protein binding: 99.6%–99.7% for alectinib and 99.1%–99.5% for M4), we understood that M4 contributed to the *in vivo* pharmacological activity of alectinib. This is consistent with the >25% criteria for the potential contribution of metabolites to pharmacological activity in the index proposed by Leclercq et al. [24, 25].

This quantitative analysis of metabolites and their contribution to pharmacological activity was essential when predicting clinical drug-drug interactions for alectinib and designing clinical studies. The information that M4 is a major metabolite of alectinib and similarly active was utilized when drug-drug interactions between alectinib and a strong CYP3A inhibitor (posaconazole) and a CYP3A inducer (rifampin) were assessed [26]. Data on the exposure of alectinib and M4 in human were used to set dosing recommendations that ensured inhibitors and inducers had minimal effect on the combined molar exposure of alectinib and M4.

## 5 Conclusion

This report describes the method by which we sufficiently identified all alectinib metabolites (M4, M6, M1b, and M1a) in human. From the analysis, the biotransformation from alectinib to M4 and M1b was found to represent two main metabolic pathways for alectinib, with M4 being the only major active metabolite that required monitoring in clinic.

As both alectinib and M4 are similarly activie in both enzyme and cell based in vitro pharmacology assays against the target ALK and exhibit similar plasma protein binding, both substances are expected to contribute to overall alectinib efficacy and safety. Therefore, the combined total exposure of alectinib and M4 was applied in the design and interpretation of clinical pharmacology studies for alectinib.

The present study has demonstrated that, in addition to the human ADME study conducted in the early phase, definitive identification of metabolites in human and evaluation of their pharmacological activities are crucial to appropriately and effectively assess the exposure and response relationship during clinical development.

## Declarations

### Author contribution statement

Mika Sato-Nakai: Conceived and designed the experiments; Performed the experiments; Analyzed and interpreted the data; Contributed reagents, materials, analysis tools or data; Wrote the paper.

Kosuke Kawashima, Toshito Nakagawa, Li Yu: Conceived and designed the experiments; Wrote the paper.

Yukako Tachibana, Miyuki Yoshida, Kenji Takanashi, Martin Binder: Performed the experiments; Analyzed and interpreted the data.

Peter N. Morcos, David J Moore: Conceived and designed the experiments.

### Funding statement

This research did not receive any specific grant from funding agencies in the public, commercial, or not-for-profit sectors.

### Competing interest statement

The authors declare no conflict of interest.

### Additional information

The clinical trial described in this paper was registered at https://clinicaltrials.gov under the registration number NCT01981005.
